# A Latent Profile Analysis of Psychological, Personality, and Religious Characteristics in Iranian Adults With Chronic Pain

**DOI:** 10.1155/prm/9958741

**Published:** 2026-06-29

**Authors:** Farzin Bagheri Sheykhangafshe, Hojjatollah Farahani, Peter Watson, Nataša Kovač

**Affiliations:** ^1^ Department of Psychology, Faculty of Humanities, Tarbiat Modares University, Tehran, Iran, modares.ac.ir; ^2^ MRC Cognition and Brain Sciences Unit, Cambridge University, 15 Chaucer Road, Cambridge, CB2 7EF, UK, cam.ac.uk; ^3^ Faculty of Applied Sciences, University of Donja Gorica, Oktoih 1, Podgorica, 81000, Montenegro, udgedu.me

**Keywords:** chronic pain, latent profile analysis, psychological features, youth

## Abstract

**Objective:**

This study aimed to identify distinct psychological profiles among individuals with chronic pain in Iran using latent profile analysis (LPA) and to examine the role of psychological, personality, and religious characteristics in the experience and management of chronic pain.

**Methods:**

The study population consisted of male and female patients with chronic pain in Tehran Province in 2024. A total of 541 patients with chronic pain (307 females and 234 males) were selected through convenience sampling based on the study’s inclusion and exclusion criteria. Data collection involved using questionnaires on demographic characteristics, grading the severity of chronic pain, temperament and character, post‐traumatic stress, learned helplessness, alexithymia, depression, anxiety and stress, pain catastrophizing, and religious orientation. Data analysis was performed using SPSS 27 and R Version 4.4.1.

**Results:**

Three latent profiles of Iranian adults with chronic pain were identified. Profile 1 (*n* = 257), moderate pain intensity/psychologically vulnerable, showed elevated psychological distress, catastrophizing, helplessness, and lower religious orientation. Profile 2 (*n* = 204), low pain intensity/balanced and religious, demonstrated the lowest levels of pain and psychological difficulties alongside higher religious orientation and character scores. Profile 3 (*n* = 80), high pain intensity/relatively resilient, reported the highest pain intensity but comparatively moderate psychological difficulties, suggesting greater resilience despite severe pain.

**Conclusion:**

The results highlight that psychological, personality, and religious characteristics play a significant role in the experience and management of chronic pain. These findings could help in developing personalized treatment strategies and improving the mental health and quality of life of patients.

## 1. Introduction

Chronic pain represents a multifaceted biopsychosocial phenomenon with profound implications for public health, functioning, and quality of life [1]. It is one of the most frequent reasons for seeking medical care and contributes substantially to disability, psychological comorbidities, and social burden [2]. According to the Global Burden of Disease studies, chronic pain—particularly musculoskeletal conditions—ranks among the top causes of long‐term disability and work inactivity worldwide, following lower back pain, depression, cancer, and heart disease [3]. Consequently, recognition of pain relief as a fundamental human right underscores the urgency of exploring comprehensive, interdisciplinary approaches to pain management [4]. Although chronic pain rarely threatens life directly, it imposes pervasive suffering that disrupts daily functioning and emotional well‐being [5, 6]. The biopsychosocial model posits that chronic pain emerges from dynamic interactions among biological, psychological, and social processes rather than a purely physiological disorder [7, 8]. From this perspective, chronic pain is not merely a sensory experience but an embodied psychological and social state that shapes behavior, resilience, and identity [9].

Epidemiological research consistently demonstrates the global ubiquity of chronic pain. Studies from Europe and Asia report prevalence rates near 25%–30% in adults, often higher among women and older individuals [10–12]. In Iran, for example, low back pain prevalence is estimated at 25.2%, highlighting a burgeoning national health concern [12]. Chronic pain also persists in specific clinical contexts, such as postsurgical or poststroke recovery, where it markedly reduces life quality [13, 14]. Yet beyond prevalence statistics, the persistence of pain across varied populations suggests shared underlying psychosocial mechanisms requiring systematic investigation. Emerging evidence indicates a rising prevalence of chronic pain among younger populations, challenging assumptions that pain is confined to aging or degenerative conditions. Large‐scale meta‐analyses reveal that roughly one in nine young adults globally experience chronic pain symptoms, frequently co‐occurring with anxiety, depression, sleep disturbance, and academic disengagement [15–17]. Despite this, relatively little is known about the psychological configurations that render some young adults more vulnerable to sustained pain than others.

Psychological determinants play a pivotal role in shaping pain experience and adaptation. Constructs such as pain catastrophizing, emotional dysregulation, post‐traumatic stress disorder (PTSD), alexithymia, learned helplessness, and personality traits have all been implicated in pain sensitivity, endurance, and recovery [18–24]. Furthermore, religiosity and spirituality may operate as protective factors, mitigating distress and reinforcing meaning‐making in adversity [25, 26]. These findings collectively reinforce that chronic pain cannot be understood or treated effectively without considering its deep psychological and existential dimensions. However, the majority of prior research has applied variable‐centered approaches, examining isolated predictors of pain rather than how multiple psychological characteristics co‐occur within individuals. Such approaches overlook underlying heterogeneity, potentially masking meaningful subgroups with distinct vulnerability and coping patterns. In contrast, latent profile analysis (LPA) offers a person‐centered framework suitable for uncovering unobserved configurations of psychological and emotional characteristics that differentiate pain experiences [27–29]. Evidence suggests that integrating LPA into chronic pain research can advance precision‐focused, individualized interventions rather than one‐size‐fits‐all models [30–32].

Yet, few studies have systematically employed person‐centered approaches to explore the joint interplay of emotional, cognitive, personality, and religious dimensions in chronic pain—particularly within non‐Western cultural contexts, where sociocultural meanings of pain and spirituality may differ substantially. To address this critical gap, the present study used LPA to identify distinct psychological profiles among young Iranian adults living with chronic pain. By jointly modeling psychological distress, PTSD symptoms, pain catastrophizing, alexithymia, learned helplessness, personality traits, and religious orientation, this research aims to expand the understanding of chronic pain heterogeneity and to inform culturally sensitive, person‐centered psychological interventions.

## 2. Methods

### 2.1. Study Design

This descriptive‐analytical, cross‐sectional study was conducted in Tehran Province in 2024 to investigate the psychological, personality, and religious characteristics of adults with chronic pain. A quantitative approach was adopted, and data were collected using self‐report instruments. The study design was informed by theoretical frameworks in clinical psychology and contemporary pain research. To account for heterogeneity within the sample, LPA, a person‐centered statistical technique, was employed to identify unobserved subgroups based on participants’ psychological and emotional characteristics. In contrast to variable‐centered approaches, LPA enables the identification of distinct psychological patterns among individuals, thereby allowing for more nuanced interpretations and potentially more tailored clinical implications. An a priori power analysis was conducted using G∗Power software (Version 3.1.9.2). Assuming a small‐to‐medium effect size, an alpha level of 0.05, and a statistical power of 0.80 for the planned F‐test analyses, the minimum required sample size was estimated to be approximately 600 participants.

### 2.2. Participants

**FIGURE 1:**
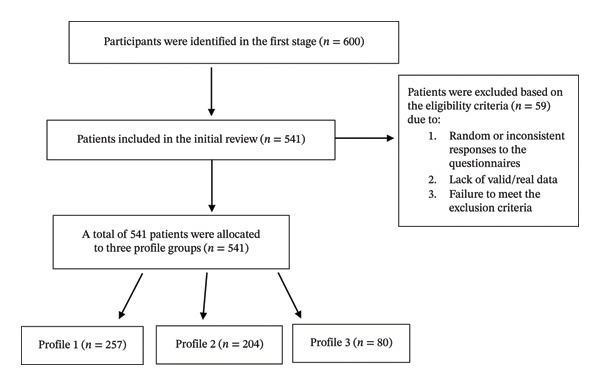
Participant flow screen diagram.

Participants were recruited through convenience sampling using both online and face‐to‐face methods. Initially, 600 individuals completed the questionnaires. After excluding 59 cases due to inconsistent, random, or invalid responses, the final sample consisted of 541 participants, including 307 women and 234 men. The mean age was 30.16 years (SD = 6.65) for women and 30.59 years (SD = 6.84) for men. The inclusion criteria were as follows: (1) self‐reported experience of chronic pain for at least six months, (2) ability and willingness to provide informed consent, (3) no current diagnosis of a severe mental disorder, and (4) not being under active psychological treatment or counseling at the time of participation. The exclusion criteria included (1) incomplete or invalid questionnaire responses and (2) exacerbation of symptoms during participation. The study was approved by the Ethics Committee of Tarbiat Modares University (IR.MODARES.REC.1401.197). All participants were fully informed about the study objectives, procedures, and potential risks, and written informed consent was obtained prior to participation. Figure 1 presents the participant selection procedure from the initial 600 individuals assessed for eligibility to the final sample of 541 participants included in the analysis, along with the reasons for exclusion.

### 2.3. Data Collection

Data collection was conducted using both online and in‐person methods to improve accessibility and accommodate the physical conditions of patients with chronic pain. The online survey was administered via Google Forms and distributed through Instagram and WhatsApp, including both public groups and private messages. Participation was voluntary, and a convenience sampling approach was used; eligible individuals were invited to complete the survey and were also encouraged to share the survey link with others who met the study inclusion criteria. In addition, face‐to‐face data collection was conducted in collaboration with pain clinics, rehabilitation centers, and physiotherapy centers in Tehran Province. With the relevant approvals, the printed questionnaires were given to suitable participants. Respondents were given adequate time and privacy to fill out the forms and were advised to answer the questions thoughtfully to guarantee the accuracy and reliability of the data. In appreciation of their participation, respondents received small monetary, symbolic, or psychological incentives. Moreover, interested participants provided a summary of the findings through email. Throughout the process, confidentiality and data protection requirements were strictly complied with (see Figure 1).

### 2.4. Measures

The Graded Chronic Pain Scale (GCPS), developed by Von Korff, Ormel, Keefe, and Dworkin [33], has been widely validated and applied in both clinical and research settings. This seven‐item instrument assesses pain intensity, pain stability or duration, and the resulting degree of disability. It uses an 11‐point numerical rating scale ranging from 0 (*no pain*) to 10 (*worst possible pain*). Participant scores are derived from three subscales: Pain Intensity, Disability Score, and Levels or Grades of Disability [33]. In the current sample, the GCPS demonstrated good internal consistency, with a Cronbach’s alpha of 0.88.

The Depression, Anxiety, and Stress Scale (DASS‐21) is the short form of the Psychological Distress Scale. It contains 21 items divided into three subscales: Depression, Anxiety, and Stress. Participants are asked to rate the extent to which they experienced each state over the past week using a 4‐point intensity or frequency scale. Lovibond and Lovibond [34] reported Cronbach’s alpha coefficients of 0.93 for the entire scale and 0.88, 0.82, and 0.90 for the depression, anxiety, and stress subscales, respectively. In the present examination, Cronbach’s alpha coefficients for depression, anxiety, and stress were 0.84, 0.83, and 0.85, respectively.

The Perth Alexithymia Questionnaire (PAQ), developed by Preece, Becerra, Robinson, Dandy, and Allan [35], is a self‐report instrument consisting of 24 items divided into five subscales. It is designed to assess difficulties in identifying and expressing emotions, as well as externally oriented thinking, key components associated with alexithymia. Participants rate each item on a 7‐point Likert scale ranging from 1 (*strongly disagree*) to 7 (*strongly agree*), yielding total scores between 24 and 168. Higher scores indicate greater challenges in recognizing and describing emotions. The subscales include Negative‐Difficulty Identifying Feelings (Items 2, 8, 14, 20), Positive‐Difficulty Identifying Feelings (Items 5, 11, 17, 23), Negative‐Difficulty Describing Feelings (Items 1, 7, 13, 19), Positive‐Difficulty Describing Feelings (Items 4, 10, 16, 22), and General‐Externally Oriented Thinking (Items 3, 6, 9, 12, 15, 18, 21, 24). The validity of the questionnaire was established through concurrent validity analysis, demonstrating that individuals reporting higher levels of alexithymia also exhibited greater difficulties in emotion regulation and higher levels of psychological distress. Additionally, the internal consistency of the questionnaire was assessed using Cronbach’s alpha, resulting in strong values ranging from 0.89 to 0.91 for all subscales, as found in the study by Preece et al. [35]. In the study by Sheykhangafshe, Farahani, and Watson [36] on an Iranian sample, the results of the bootstrapped exploratory graph analysis (EGA) revealed a two‐dimensional structure for the PAQ. Factor 1 was identified as Negative Difficulty in describing and Identifying Feelings, while Factor 2 was characterized as General‐Externally Oriented Thinking. This configuration demonstrated strong structural integrity and item consistency. In the present study, the total PAQ score demonstrated excellent internal consistency, with a Cronbach’s alpha of 0.91.

The Pain Catastrophizing Scale (PCS) was developed by Sullivan, Bishop, and Pivik in 1995 to assess various aspects of pain catastrophizing and to gain a better understanding of how it affects the pain experience [37]. This questionnaire consists of 13 items, each rated on a 5‐point Likert scale. Participants are asked to choose a number from 0 (*never*) to 4 (*always*) to indicate how often they experience 13 different feelings and thoughts related to pain. Factor analysis has shown that catastrophizing involves three subscales: rumination, magnification, and helplessness. In the present study, the PCS demonstrated good internal consistency, with a Cronbach’s alpha of 0.83.

The Post‐Traumatic Stress Disorder Checklist (PCL‐5): It was developed based on the Diagnostic and Statistical Manual of Mental Disorders, Fifth Edition (DSM‐5), and serves as a self‐report measure to screen individuals for PTSD [38]. This checklist consists of 20 items divided into five subscales: re‐experiencing, avoidance, negative alterations in cognition and mood, hyperarousal, and emotional numbness. Participants rate each item on a Likert scale from 0 (*not at all* or *only one time*) to 3 (*five or more times a week* or *almost always*), resulting in total scores ranging from 0 to 80. A score of 50 or above is considered the cutoff point for a PTSD diagnosis. The reliability of the checklist is supported by Cronbach’s alpha coefficients and test–retest reliability measures, both exceeding 0.70 for the overall scale and its dimensions, indicating satisfactory reliability [38]. In this study, the PCL‐5 exhibited strong internal consistency, with a Cronbach’s alpha of 0.90.

Temperament and Character Inventory (TCI), created by Cloninger et al. in 1994 [39], is a comprehensive tool designed to assess dimensions of temperament and character through 125 items with binary response options (true/false). The TCI is divided into two major components: temperament, consisting of four subscales (harm avoidance, novelty seeking, reward dependence, and persistence), and character, consisting of three subscales (self‐directedness, cooperativeness, and self‐transcendence). Higher scores in each subscale reflect stronger tendencies in the respective trait. In the present study, Cronbach’s alpha coefficients were as follows: total score (0.76), novelty seeking (0.80), harm avoidance (0.92), reward dependence (0.90), persistence (0.78), self‐directedness (0.77), cooperativeness (0.92), and self‐transcendence (0.86). These results indicate acceptable to excellent internal consistency, reinforcing the reliability of the instrument in assessing psychological traits related to chronic pain. In the present study, the TCI demonstrated good internal consistency, with Cronbach’s alpha values of 0.81 for temperament and 0.84 for character.

Religious Orientation Scale (ROS): Originally introduced by Allport and Ross [40], this questionnaire comprised a 21‐item scale, with 11 items on external religious orientation and 9 items focusing on internal religious orientation. Subsequently, in 1963, Feagin presented a 12‐item version by incorporating an additional option highly correlated (0.61) with extrinsic orientation. As a result, the first 12 items assess external religious orientation, while the remaining 9 items evaluate internal religious orientation. Scoring for all questionnaire items followed this pattern: *strongly disagree* (5), *disagree* (4), *agree* (2), and *strongly agree* (1). Lower scores indicated individuals with internal religious orientation, while higher scores reflected external religious orientation. Allport and Ross reported Cronbach’s alpha coefficients of 0.79 and 0.82 for external religious orientation and internal religious orientation, respectively. The test–retest reliability of this scale was determined to be 0.73 after one month [40]. In the present study, Cronbach’s alpha coefficient for the total score (0.89) and subscales of external religious orientation (0.81) and internal religious orientation (0.83) were obtained.

Learned Helplessness Questionnaire (LHQ): Developed by Quinless & Nelson in 1988, this questionnaire comprises 20 Likert‐type items offering 4 response options, with scoring ranging from 1 (*strongly disagree*) to 4 (*strongly agree*). Quinless & Nelson [41] documented the questionnaire’s validity as 0.79 and its reliability, along with a Cronbach’s alpha of 0.86. Upon assessing the original version of the scale alongside other existing questionnaires like the Beck Depression Inventory, a correlation coefficient of 0.25 was noted. Compared to the Rosenberg Self‐Esteem Scale, a correlation coefficient of 0.62 was observed, with a reliability of 0.85. Utilizing Varimax rotation and exploratory factor analysis, five primary factors emerged: internal‐external, stability‐instability, general‐specific, control‐helplessness, and personal selection conditions in situations where individuals deliberately participate [41]. The present study reported a Cronbach’s alpha coefficient of 0.84.

### 2.5. Data Analysis

Data analysis was carried out using SPSS 27 and R V.4.4.1. Descriptive statistics (frequencies, percentages, means, and standard deviations) were used to summarize participant characteristics. Inferential analyses included univariate and multivariate analysis of variance (ANOVA and MANOVA), as well as logistic regression and other statistical procedures appropriate to the nature of the data. LPA was employed to identify subgroups within the sample based on shared psychological and personality features. Model selection was guided by statistical fit indices such as the Bayesian information criterion (BIC), Akaike information criterion (AIC), entropy, and the Lo–Mendell–Rubin adjusted likelihood ratio test (LMR‐LRT). These analyses provided insight into latent patterns of psychological responses and supported the development of clinically meaningful profiles.

Missing values across all variables on the 541 youths in this study were carefully examined. Missing data were minimal (< 5%) and addressed using multiple imputation (the MICE package in R) to ensure that the analyses were not biased by incomplete responses. This approach preserves statistical power and provides valid estimates, ensuring that the results are robust and reliable.

## 3. Results

In this study, 541 Iranian youths with chronic pain were assessed. The sample comprised 56.7% females and 43.3% males, with 53.2% married and 46.8% single. Most participants were aged 27–36 (45.9%), followed by 18–26 (34.5%) and 37–45 (19.6%). In terms of education, the majority held a bachelor’s degree (29%) or an associate’s degree (28.1%), while 23.3% had a master’s, 10.5% a high school diploma, and 9.1% a doctorate. Regarding pain duration, 27.7% reported experiencing chronic pain for 2 years, 20% for 3 years, and 31.4% for 4–5 years. The most common types of chronic pain were neck pain (35.3%), low back pain (21.3%), and headache (16.6%), with smaller proportions reporting fibromyalgia (11.5%), rheumatoid arthritis (9.4%), and pelvic pain (5.9%). Table 1 presents the frequency and percentage distributions of the participants’ demographic characteristics, categorized across the three identified latent profiles. The associations between demographic variables and latent profiles were examined using the chi‐square test.

**TABLE 1:** Demographic information of participants.

Variable	Profile 1	Profile 2	Profile 3	Chi‐square (*χ* ^2^)	*p* value
Gender				1.67	0.55
Female, *n* (%)	149 (58%)	110 (53.9%)	48 (60%)		
Male, *n* (%)	108 (42%)	94 (46.1%)	32 (40%)		
Marital status				0.69	0.76
Single, *n* (%)	116 (45.1%)	99 (48.5%)	38 (47.5%)		
Married, *n* (%)	141 (54.9%)	105 (51.5%)	42 (52.5%)		
Education level				9.19	0.415
Diploma, *n* (%)	22 (8.6%)	25 (12.3%)	10 (12.5%)		
Associate degree, *n* (%)	80 (31%)	57 (27.9%)	15 (18.8%)		
Bachelor’s, *n* (%)	77 (30%)	55 (27%)	25 (31.3%)		
Master’s, *n* (%)	58 (22.5%)	49 (24%)	19 (23.8%)		
Doctorate, *n* (%)	20 (7.8%)	18 (8.8%)	11 (13.8%)		
Duration of pain				31.55	*p* < 0.01
2 years, *n* (%)	81 (31.5%)	52 (25.5%)	17 (21.3%)		
3 years, *n* (%)	52 (20.2%)	35 (17.2%)	21 (26.3%)		
4 years, *n* (%)	36 (14%)	32 (15.7%)	19 (23.8%)		
5 years, *n* (%)	45 (17.4%)	31 (15.2%)	7 (8.8%)		
6 years, *n* (%)	29 (11.3%)	29 (14.2%)	5 (6.3%)		
7 years, *n* (%)	5 (1.9%)	9 (4.4%)	1 (1.3%)		
8 years, *n* (%)	3 (1.2%)	13 (6.4%)	7 (8.8%)		
9 years, *n* (%)	6 (2.3%)	3 (1.5%)	3 (3.8%)		
Type of chronic pain				14.91	0.135
Low back pain, *n* (%)	62 (24.1%)	38 (18.6%)	15 (18.8%)		
Neck pain, *n* (%)	91 (35.3%)	71 (34.8%)	29 (36.3%)		
Headache, *n* (%)	43 (16.7%)	38 (18.6%)	9 (11.3%)		
Fibromyalgia, *n* (%)	27 (10.5%)	21 (10.3%)	14 (17.5%)		
Rheumatoid arthritis, *n* (%)	24 (9.3%)	23 (11.3%)	4 (5%)		
Pelvic pain, *n* (%)	10 (3.9%)	13 (6.4%)	9 (11.3%)		

Based on Table 1, the chi‐square test results indicated that Profiles 1, 2, and 3 did not differ significantly in terms of gender (*χ* ^2^ = 1.67), marital status (*χ* ^2^ = 0.54), education level (*χ* ^2^ = 9.8), and type of chronic pain (*χ* ^2^ = 14.91), with all *p* values greater than 0.05 (*p* > 0.05). However, a significant difference was found among the profiles regarding the duration of chronic pain (*χ* ^2^ = 31.55, *p* < 0.05). The significant chi‐square for duration of pain means that the distribution of how long participants have been experiencing pain differs systematically between the three profiles. In other words, certain profiles are more likely to have participants with longer or shorter pain durations than others.

As indicated in Table 2, the three identified profiles differed significantly in chronic pain intensity and related psychological characteristics. Profile 3 (high pain intensity/relatively resilient) demonstrated the highest mean chronic pain scores, followed by Profile 1 (moderate pain intensity/psychologically vulnerable), whereas Profile 2 (low pain intensity/balanced and religious) showed the lowest mean pain scores. These findings suggest that the profiles represent distinct levels of chronic pain experience accompanied by different psychological patterns. Regarding psychological distress indicators, Profile 1 exhibited the highest mean scores in PTSD, alexithymia, pain catastrophizing, psychological distress, and learned helplessness, reflecting substantial psychological vulnerability. Profile 3 showed moderate levels on these variables, whereas Profile 2 consistently demonstrated the lowest levels of distress‐related characteristics.

**TABLE 2:** Means and standard deviations of variables by profile (*N* = 541).

Variable	Profile 1 (*n* = 257)		Profile 2 (*n* = 204)		Profile 3 (*n* = 80)	
	Mean (M)	SD	Mean (M)	SD	Mean (M)	SD
Chronic pain	39.07	5.94	17.02	6.44	44.07	7.78
Post‐traumatic stress disorder	62.89	9.05	39.23	10.29	50.35	13.50
Alexithymia	103.64	14.87	60.51	17.51	76.41	26.17
Pain catastrophizing	27.85	5.94	12.49	5.23	17.75	8.59
Psychological distress	52.81	6.16	31.25	9.26	36.11	8.59
External religious orientation	21.33	4.30	36.26	6.90	27.73	5.20
Internal religious orientation	17.29	3.82	26.74	3.69	23.12	4.46
Temperament	99.50	4.63	94.96	6.05	98.92	7.83
Character	56.82	11.87	90.21	9.76	83.75	14.96
Learned helplessness	73.95	9.65	44.90	10.89	54.51	14.40

**FIGURE 2:**
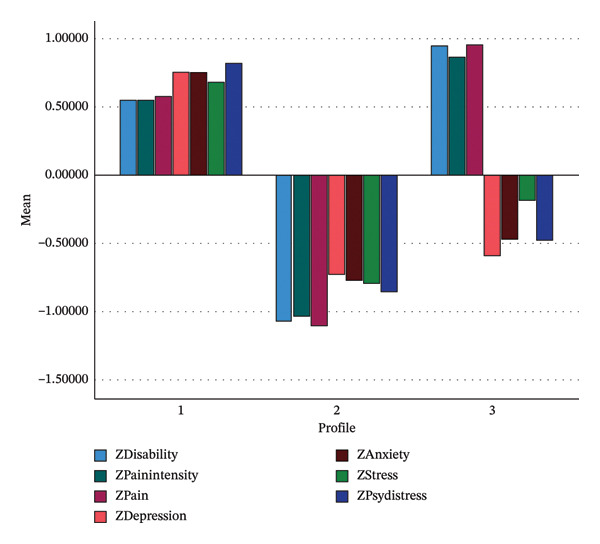
Comparison of chronic pain and psychological distress among Profiles 1, 2, and 3.

Differences were also observed in religious orientation and personality dimensions. Profile 2 reported the highest levels of both internal and external religious orientation, followed by Profile 3, while Profile 1 showed the lowest levels. In terms of temperament, Profile 1 demonstrated the highest mean scores, followed by Profile 3, whereas Profile 2 showed the lowest temperament scores. Conversely, character traits were highest in Profile 2, moderate in Profile 3, and lowest in Profile 1. Overall, LPA identified three distinct subgroups among the 541 Iranian participants with chronic pain. Profile 1 (moderate pain intensity/psychologically vulnerable; *n* = 257) was characterized by elevated psychological distress and maladaptive cognitive‐emotional features. Profile 2 (low pain intensity/balanced and religious; *n* = 204) demonstrated the lowest levels of psychological distress alongside the highest levels of religious orientation and character strengths, suggesting greater psychological resilience. Profile 3 (high pain intensity/relatively resilient; *n* = 80) showed the highest pain intensity but only moderate levels of psychological distress, indicating relatively adaptive functioning despite severe pain. Figure 2 illustrates the differences among these profiles in chronic pain intensity and psychological characteristics.

To clarify the rationale for selecting the optimal number of latent profiles, several model fit indices and likelihood ratio tests were examined (see Table 3). Model fit was evaluated using AIC, BIC, KIC, SABIC, AWE, and CLC, with lower values indicating better fit. Entropy was considered as indicator of classification accuracy, with higher values reflecting clearer separation between profiles. In addition, the Bootstrapped Likelihood Ratio Test (BLRT) was used to determine whether adding an additional profile significantly improved model fit. The BLRT results showed that moving from the one‐profile to the two‐profile solution (*p* = 0.010) and from the two‐profile to the three‐profile solution (*p* = 0.010) resulted in significant improvements in model fit. However, the addition of a fourth profile to the three‐profile model was not statistically significant (*p* = 0.188), suggesting that the four‐profile solution did not meaningfully improve upon the three‐profile model.

**TABLE 3:** Fit indices and likelihood ratio tests for different models in LPA.

Model	AIC	BIC	AWE	CLC	KIC	SABIC	BLRT	Entropy
Profile 1	6657.44	6678.90	6723.37	6649.44	6665.44	6663.03	—	1.00
Profile 2	6480.13	6527.36	6627.75	6459.97	6494.13	6492.44	0.010	0.918
Profile 3	**6452.99**	**6525.98**	**6682.67**	**6420.28**	**6472.99**	**6472.01**	**0.010**	**0.646**
Profile 4	6455.23	6553.98	6752.45	6410.50	6481.23	6480.97	0.188	0.637
Profile 5	6359.44	6483.95	6752.12	6302.77	6391.44	6391.89	0.010	0.666

Although the five‐profile solution yielded the lowest AIC value, model selection was not based solely on statistical fit. The five‐profile model showed only marginal improvements in some indices and introduced greater complexity with limited theoretical interpretability. In contrast, the three‐profile solution demonstrated an appropriate balance between model fit (AIC = 6452.99; BIC = 6525.98), acceptable classification accuracy (entropy = 0.646), parsimony, and conceptual clarity. Taken together, these findings indicate that the three‐profile (*k* = 3) solution, highlighted in bold in Table 3, provides the most theoretically meaningful and statistically defensible representation of heterogeneity in youth with chronic pain, and it was therefore selected as the final model.

Prior to conducting the multinomial logistic regression analysis, a one‐way ANOVA was performed to examine differences across the identified profiles. The results indicated significant differences among the profiles, providing justification for further investigation of the predictors of profile membership. As presented in Table 4, the multinomial logistic regression results showed that several psychological and personality variables significantly predicted latent profile membership among youth with chronic pain. Learned helplessness emerged as a strong predictor: each one‐unit increase increased the odds of belonging to Profile 1 (moderate pain intensity/psychologically vulnerable) by 1.17 times and Profile 3 (high pain intensity/relatively resilient) by 1.06 times (both *p* < 0.001). Similarly, higher chronic pain scores significantly increased the likelihood of membership in Profiles 1 and 3 compared with Profile 2 (low pain intensity/balanced and religious), with odds ratios of 1.68 and 1.88, respectively.

**TABLE 4:** Multinomial logistic regression results predicting profiles based on variables.

Variable	Comparison (predictors)	Coefficient (B)	Std. error	Wald statistic	Odds ratio (exp[B])	95% CI for odds ratio	*p* value
Chronic pain	Profile 1 vs. Profile 2	0.519	0.063	67.05	1.68	1.48–1.90	< 0.001
	Profile 3 vs. Profile 2	0.635	0.067	89.90	1.88	1.65–2.15	< 0.001

PTSD	Profile 1 vs. Profile 2	0.188	0.014	168.46	1.20	1.17–1.24	< 0.001
	Profile 3 vs. Profile 2	0.092	0.013	49.23	1.09	1.07–1.12	< 0.001

Alexithymia	Profile 1 vs. Profile 2	0.110	0.008	173.27	1.11	1.10–1.13	< 0.001
	Profile 3 vs. Profile 2	0.037	0.007	29.64	1.04	1.02–1.05	< 0.001

Pain catastrophizing	Profile 1 vs. Profile 2	0.330	0.025	170.30	1.39	1.32–1.46	< 0.001
	Profile 3 vs. Profile 2	0.128	0.022	132.84	1.13	1.08–1.18	< 0.001

Psychological distress	Profile 1 vs. Profile 2	0.570	0.056	103.60	1.76	1.58–1.76	< 0.001
	Profile 3 vs. Profile 2	0.074	0.020	14.45	1.07	1.03–1.07	< 0.001

Internal orientation	Profile 1 vs. Profile 2	−0.442	0.033	179.59	0.64	0.60–0.68	< 0.001
	Profile 3 vs. Profile 2	−0.183	0.031	34.54	0.83	0.78–0.88	< 0.001

External orientation	Profile 1 vs. Profile 2	−0.370	0.029	158.79	0.69	0.65–0.73	< 0.001
	Profile 3 vs. Profile 2	−0.162	0.021	57.12	0.85	0.81–0.88	< 0.001

Temperament	Profile 1 vs. Profile 2	0.144	0.019	56.14	1.15	1.11–1.19	< 0.001
	Profile 3 vs. Profile 2	0.127	0.025	26.45	1.13	1.08–1.19	< 0.001

Character	Profile 1 vs. Profile 2	−0.151	0.012	171.49	0.86	0.84–0.87	< 0.001
	Profile 3 vs. Profile 2	−0.042	0.011	15.08	0.96	0.94–0.98	< 0.001

Learned helplessness	Profile 1 vs. Profile 2	0.162	0.012	180.41	1.17	1.14–1.20	< 0.001
	Profile 3 vs. Profile 2	0.060	0.011	31.68	1.06	1.04–1.08	< 0.001

**FIGURE 3:**
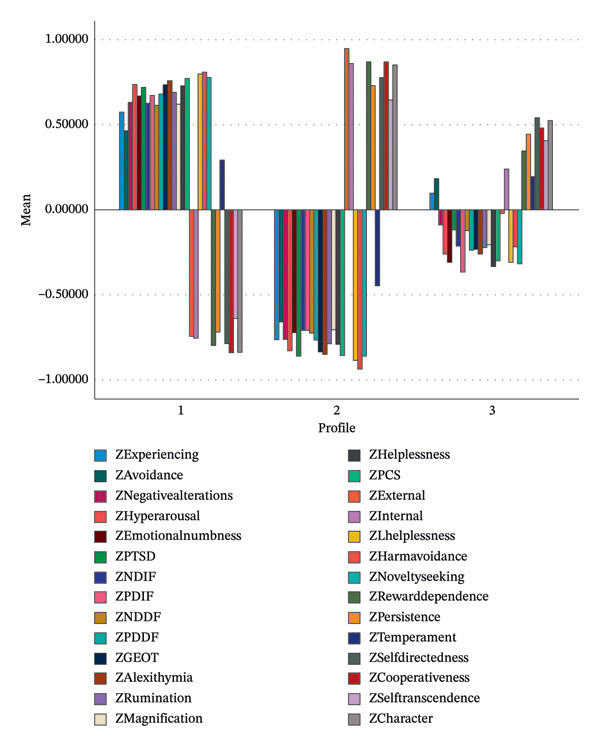
Comparison of psychological characteristics among Profiles 1, 2, and 3.

Additional psychological factors—including PTSD symptoms, alexithymia, pain catastrophizing, and overall psychological distress—also significantly increased the probability of classification into Profiles 1 and 3, highlighting the important role of psychological burden in these groups. In contrast, both internal and external religious orientations were negatively associated with Profiles 1 and 3, indicating that higher levels of religiosity were linked with a greater likelihood of membership in Profile 2, the more psychologically balanced and resilient group. Furthermore, temperament positively predicted membership in Profile 1, whereas higher character scores increased the likelihood of belonging to Profile 2, underscoring the contribution of personality dimensions to the differentiation of latent profiles. The pattern of variable scores across these profiles is also illustrated in Figure 3.

## 4. Discussion

The present study aimed to identify distinct psychological profiles among Iranian adults living with chronic pain using a person‐centered analytic approach. The findings revealed three meaningful profiles that differed not only in pain intensity but also in emotional functioning, cognitive responses to pain, personality structure, and religious orientation. These patterns highlight that chronic pain is not merely a physical condition but a complex biopsychosocial experience shaped by the interaction of psychological vulnerabilities and protective resources. Identifying such profiles provides a deeper understanding of the heterogeneous ways individuals experience and cope with chronic pain and underscores the importance of tailored psychological interventions.

The first profile, moderate pain intensity/psychologically vulnerable, represents individuals whose experience of pain is strongly intertwined with emotional dysregulation and maladaptive cognitive processes. Although their physical pain is not the most severe, this group exhibits the most pronounced psychological difficulties, including heightened emotional distress, trauma‐related symptoms, difficulties in identifying and expressing emotions, and a tendency toward catastrophic interpretations of pain [31]. Such a constellation suggests that pain in this group is embedded within a broader psychological vulnerability characterized by negative affectivity, cognitive rigidity, and diminished emotional processing capacity [27]. These mechanisms may intensify the subjective experience of pain by directing attention toward bodily discomfort and reinforcing beliefs that pain is overwhelming and uncontrollable [20]. From a psychological perspective, this profile reflects a maladaptive cycle in which emotional distress, catastrophic thinking, and learned helplessness mutually reinforce one another. Individuals may perceive themselves as lacking control over their symptoms and may struggle to mobilize adaptive coping strategies [16]. Furthermore, relatively weaker spiritual orientation and lower character development may limit access to meaning‐based coping resources that often help individuals contextualize and tolerate suffering. Consequently, the experience of pain becomes psychologically amplified, producing a pattern in which emotional vulnerability magnifies physical discomfort [9]. Clinical interventions for this group may therefore need to prioritize emotional regulation, cognitive restructuring, and the development of adaptive coping and meaning‐making strategies [14].

The second profile, low pain intensity/balanced and religious, reflects a pattern of psychological balance and adaptive coping. Individuals in this group report relatively low levels of emotional distress, limited engagement in catastrophic thinking, and fewer trauma‐related symptoms, suggesting a stable emotional environment in which pain does not dominate psychological functioning [10]. Their psychological profile appears characterized by effective emotional regulation, cognitive flexibility, and a reduced tendency to interpret physical discomfort as threatening or overwhelming [32]. Such patterns indicate the presence of adaptive psychological processes that prevent the escalation of pain into broader emotional suffering. A defining feature of this profile is the strong presence of religious orientation and well‐developed character strengths [24]. Spiritual beliefs and practices may provide individuals with existential meaning, emotional reassurance, and culturally meaningful frameworks for understanding adversity [29]. Through these frameworks, pain can be interpreted not merely as a source of suffering but as an experience that may carry personal, moral, or spiritual significance [42]. These interpretive processes may reduce emotional reactivity and foster acceptance, patience, and hope. Consequently, this profile appears to represent a psychologically protected group in which spiritual resources and personality strengths function together as buffers against the emotional burden of chronic pain [16].

The third profile, high pain intensity/relatively resilient, illustrates a different pattern in which individuals experience severe physical pain yet maintain comparatively stable psychological functioning. Unlike the psychologically vulnerable group, individuals in this profile do not exhibit extreme levels of emotional distress, catastrophic thinking, or helplessness. Instead, their psychological responses appear relatively moderated, suggesting that the presence of intense physical symptoms does not necessarily translate into profound psychological impairment [20]. This distinction highlights the important role of psychological resources in shaping the subjective impact of chronic pain. The relative psychological stability observed in this group may reflect the presence of adaptive coping strategies, personality strengths, and moderate spiritual resources that enable individuals to maintain functional equilibrium despite persistent pain [26]. Such individuals may possess greater tolerance for discomfort, stronger problem‐solving capacities, or more effective emotional regulation skills, which allow them to manage pain without becoming overwhelmed by it [43]. In this sense, the profile reflects a form of psychological resilience in which individuals continue to adapt and function even under conditions of substantial physical strain [9]. Interventions targeting this group may therefore benefit from reinforcing existing coping capacities and supporting the maintenance of resilience in the face of ongoing pain [27].

Despite the contributions of the present study to understanding psychological profiles among Iranian adolescents and young adults with chronic pain, several limitations should be considered. First, the sample was limited to residents of Tehran Province, which may restrict the generalizability of the findings to other cultural and regional contexts within Iran. Second, the use of purposive sampling may have introduced selection bias and limited sample representativeness. In addition, although validated self‐report measures were used, the findings may have been influenced by social desirability and recall biases. The study also did not directly examine the role of cultural and ethnic diversity in shaping the perception and expression of chronic pain. Finally, the cross‐sectional design prevents conclusions regarding causality or temporal relationships between psychological characteristics and pain outcomes. Future studies should recruit more diverse and representative samples from different regions and cultural backgrounds and employ longitudinal designs to better understand changes in psychological functioning over time. Furthermore, the use of mixed‐methods approaches and greater attention to cultural and spiritual dimensions of chronic pain may provide a more comprehensive understanding of individuals’ lived experiences and coping processes.

## 5. Conclusion

Overall, the findings of this study underscore the heterogeneous and multidimensional nature of chronic pain by demonstrating that individuals with similar physical symptoms may experience profoundly different psychological realities. The three identified profiles suggest that the burden of chronic pain is not determined solely by pain intensity but rather by the dynamic interplay between emotional regulation, cognitive appraisal, personality characteristics, and spiritual resources. In particular, the results highlight that psychological vulnerability—manifested through emotional distress, maladaptive pain cognitions, and diminished coping resources—can amplify the subjective impact of pain even when physical intensity is not at its highest. Conversely, the presence of adaptive psychological and spiritual resources appears capable of buffering the emotional consequences of chronic pain and supporting more balanced adjustment. These findings emphasize the importance of adopting a person‐centered perspective in chronic pain research and clinical practice, as different subgroups may require fundamentally different therapeutic approaches. Interventions that focus on improving emotional awareness, reducing catastrophic thinking, strengthening adaptive coping, and facilitating meaning‐making processes may be particularly beneficial for psychologically vulnerable individuals, whereas resilience‐oriented and strength‐based strategies may help maintain adaptive functioning among those who remain psychologically stable despite severe pain. Taken together, the identified profiles highlight the critical role of psychological and spiritual factors in shaping the lived experience of chronic pain and point toward the necessity of integrative, individualized approaches in both assessment and treatment.

## Author Contributions

Farzin Bagheri Sheykhangafshe contributed to the study conception, design, and data collection. Hojjatollah Farahani performed the data analysis and interpretation. Peter Watson and Nataša Kovač contributed to drafting and revising the manuscript.

## Funding

No funding was received for this manuscript.

## Disclosure

All authors reviewed and approved the final version of the manuscript.

## Conflicts of Interest

The authors declare no conflicts of interest.

## Supporting Information

Additional supporting information can be found online in the Supporting Information section.

## Supporting information


**Supporting Information** A STROBE checklist is provided.

## Data Availability

The datasets generated and analyzed during the current study are not publicly available due to confidentiality agreements with participants and ethical considerations but are available from the corresponding author upon reasonable request and with appropriate institutional approvals.

## References

[1] Edwards K. A. , You D. S. , Lannon E. W. , Dildine T. C. , Darnall B. D. , and Mackey S. C. , Beyond Pain Intensity: Validating single-item Pain Bothersomeness Measures, The Journal of Pain. (2025) 31, 10.1016/j.jpain.2025.105395. PMC12603663 40228688

[2] Dassieu L. , Heino A. , Develay É. et al., Conversations About Opioids: Impact of the Opioid Overdose Epidemic on Social Interactions for People who Live with Chronic Pain, Qualitative Health Research. (2021) 31, no. 9, 1657–1669, 10.1177/10497323211005829. 33834915

[3] Zhu M. , Zhang J. , Liang D. et al., Global and Regional Trends and Projections of Chronic Pain from 1990 to 2035: Analyses Based on Global Burden of Diseases Study 2019, British Journal of Pain. (2024) 19, no. 2, 125–137, 10.1177/20494637241310697. 39726775 PMC11669129

[4] Nelson E. U. , Social Determinants of Chronic Pain Management for People Who Use Drugs: an Ethics of Care Approach, Nursing Inquiry. (2025) 32, no. 2, 10.1111/nin.70003. 40000920

[5] Driscoll M. A. , Edwards R. R. , Becker W. C. , Kaptchuk T. J. , and Kerns R. D. , Psychological Interventions for the Treatment of Chronic Pain in Adults, Psychological Science in the Public Interest. (2021) 22, no. 2, 52–95, 10.1177/1529100621995624. 34541967

[6] Park J. H. , Prochnow T. , Smith M. L. , and Kim S. J. , Health Disparities of Healthcare Utilization and Opioid Use Disorders Among Chronic Pain Patients: Examination of a Representative National Inpatient Sample of US Hospitals from 2016–2020, International Journal of Mental Health and Addiction. (2025) 24, no. 2, 1–16, 10.1007/s11469-025-00760-x.

[7] Nicholas M. , Vlaeyen J. W. , Rief W. et al., The IASP Classification of Chronic Pain for ICD-11: Chronic Primary Pain, Pain. (2019) 160, no. 1, 28–37, 10.1097/j.pain.0000000000001442. 30586068

[8] Raffaeli W. , Tenti M. , Corraro A. et al., Chronic Pain: what Does it Mean? A Review on the Use of the Term Chronic Pain in Clinical Practice, Journal of Pain Research. (2021) 14, 827–835, 10.2147/JPR.S297363. 33833560 PMC8019660

[9] Leake H. B. , Moseley G. L. , Murphy L. K. , Murray C. B. , Palermo T. M. , and Heathcote L. C. , How Does Pain Work? A Qualitative Analysis of How Young Adults with Chronic Pain Conceptualize the Biology of Pain, European Journal of Pain. (2023) 27, no. 3, 424–437, 10.1002/ejp.1960. 36527324 PMC10947129

[10] Thompson W. H. , Thern E. , Gedin F. , Andreasson A. , Jensen K. B. , and Lalouni M. , Early Signs of long-term Pain: Prospective Network Profiles from Late Adolescence and Lifelong follow-up, NPJ Mental Health Research. (2025) 4, no. 1, 10.1038/s44184-024-00038-x. PMC11822022 39939815

[11] Dueñas M. , De Sola H. , Salazar A. , Esquivia A. , Rubio S. , and Failde I. , Prevalence and Epidemiological Characteristics of Chronic Pain in the Spanish Population. Results from the Pain Barometer, European Journal of Pain. (2025) 29, no. 1, 10.1002/ejp.4705. PMC11609938 39046161

[12] Ghafouri M. , Teymourzadeh A. , Nakhostin-Ansari A. et al., Prevalence and Predictors of Low Back Pain Among the Iranian Population: Results from the Persian Cohort Study, Annals of Medicine and Surgery. (2022) 74, 10.1016/j.amsu.2021.103243. PMC8801351 35145656

[13] Mottet B. , Cayla C. , Bernard T. , Léger M. , Campfort M. , and Lasocki S. , Prevalence of Chronic Pain and Its Risk Factors Until One Year After Intensive Care Unit Discharge. A single-center Prospective Observational Study, Intensive and Critical Care Nursing. (2025) 89, 10.1016/j.iccn.2024.103969. 39951966

[14] Rahmatian A. , Bastani E. , Shokri F. , and Karbasfrushan A. , Prevalence of Hemiplegic Shoulder Pain in Iran: a Systematic Review and meta-analysis, Anesthesiology and Pain Medicine. (2023) 13, no. 3, 10.5812/aapm-136423. PMC10664159 38021328

[15] Murray C. B. , de la Vega R. , Murphy L. K. , Kashikar-Zuck S. , and Palermo T. M. , The Prevalence of Chronic Pain in Young Adults: a Systematic Review and meta-analysis, Pain. (2022) 163, no. 9, e972–e984, 10.1097/j.pain.0000000000002488. 34817439

[16] Wager J. , Brown D. , Kupitz A. , Rosenthal N. , and Zernikow B. , Prevalence and Associated Psychosocial and Health Factors of Chronic Pain in Adolescents: Differences by Sex and Age, European Journal of Pain. (2020) 24, no. 4, 761–772, 10.1002/ejp.1521. 31889351

[17] Shaygan M. , Jaberi A. , Razavizadegan M. , and Shayegan Z. , Prevalence of Chronic Pain and Contributing Factors: a cross-sectional Population-based Study Among 2,379 Iranian Adolescents, Korean Journal of Pain. (2023) 36, no. 2, 230–241, 10.3344/kjp.2023.36.2.230. 36973970 PMC10043785

[18] Marchi L. , Marzetti F. , Orrù G. et al., Alexithymia and Psychological Distress in Patients with Fibromyalgia and Rheumatic Disease, Frontiers in Psychology. (2019) 10, 10.3389/fpsyg.2019.01735. PMC6685004 31417462

[19] Moreno J. E. , Nestor B. A. , Mitcheson M. , and Nelson S. , The Moderating Role of Ethnicity on Depressive and Anxiety Symptoms and Pain Catastrophizing in Hispanic/Latinx and Non-Hispanic/Latinx White Youth with Chronic Pain, The Clinical Journal of Pain. (2025) 41, no. 3, 10.1097/AJP.0000000000001175. 39928544

[20] Lund T. , Bernier E. , Roman-Juan J. et al., Pain and Post-traumatic Stress Disorder Symptoms: Dyadic Relationships Between Canadian Armed Forces Members/Veterans with Chronic Pain and Their Offspring, Journal of Pain. (2024) 25, no. 8, 10.1016/j.jpain.2023.12.004. 38580101

[21] Aho T. , Sipilä R. , Kalso E. , and Harno H. , Temperament and Character Dimensions Differ in Chronic Post-surgical Neuropathic Pain and Cold Pressure Pain, Scand Journal of Pain. (2022) 22, no. 3, 515–525, 10.1515/sjpain-2021-0123. 35139264

[22] Ferreira-Valente A. , Damião C. , Pais-Ribeiro J. , and Jensen M. P. , The Role of Spirituality in Pain, Function, and Coping in Individuals with Chronic Pain, Pain Medicine. (2020) 21, no. 3, 448–457, 10.1093/pm/pnz163. 31045211

[23] Yessick L. R. and Salomons T. V. , The Chronic Disease Helplessness Survey: Developing and Validating a Better Measure of Helplessness for Chronic Conditions, Pain Reports. (2022) 7, no. 2, 10.1097/PR9.0000000000000991. PMC8923572 35311028

[24] Aaron R. V. , Preece D. A. , Heathcote L. C. , Wegener S. T. , Campbell C. M. , and Mun C. J. , Assessing Alexithymia in Chronic Pain: Psychometric Properties of the Toronto Alexithymia Scale-20 and Perth Alexithymia Questionnaire, Pain Reports. (2025) 10, no. 1, 10.1097/PR9.0000000000001204. PMC11631001 39664711

[25] Hill P. , Chronic Pain: a Consequence of Dysregulated Protective Action, British Journal of Pain. (2019) 13, no. 1, 13–21, 10.1177/2049463718825042. 30671234 PMC6327352

[26] Fang S. and Chung M. C. , Testing the Pain Paradox: a Longitudinal Study on PTSD from past Trauma, Alexithymia, Mindfulness, and Psychological Distress, Current Psychology. (2023) 42, no. 11, 8844–8854, 10.1007/s12144-021-02519-x.

[27] Slawek D. E. , Syed M. , Cunningham C. O. et al., Pain Catastrophizing and Mental Health Phenotypes in Adults with Refractory Chronic Pain: a Latent Class Analysis, Journal of Psychiatric Research. (2022) 145, 102–110, 10.1016/j.jpsychires.2021.12.018. 34890916 PMC9160202

[28] Perrin J. , Streeck N. , Naef R. , Rufer M. , Peng-Keller S. , and Rettke H. , Comparing Perspectives: Patients’ and Health Care Professionals’ Views on Spiritual Concerns and Needs in Chronic Pain care-a Qualitative Study, BMC Health Services Research. (2021) 21, no. 1, 10.1186/s12913-021-06562-1. PMC8152324 34039337

[29] Kovač N. , Ratković K. , Watson P. , Farahani H. , and Bagheri Sheykhangafshe F. , Machine Learning Classification Models for Predicting Chronic Pain, Current Psychology. (2025) 44, no. 18, 15409–15422, 10.1007/s12144-025-08294-w.

[30] Kittelson A. J. , Schmiege S. J. , Maluf K. , George S. Z. , and Stevens-Lapsley J. E. , Determination of Pain Phenotypes in Knee Osteoarthritis Using Latent Profile Analysis, Pain Medicine. (2021) 22, no. 3, 653–662, 10.1093/pm/pnaa314. 33367906 PMC7971470

[31] Kim S. , Lee J. , and Boone D. , Protective and Risk Factors at the Intersection of Chronic Pain, Depression, Anxiety, and Somatic Amplification: a Latent Profile Approach, Journal of Pain Research. (2022) 15, 1107–1121, 10.2147/JPR.S347193. 35450061 PMC9018014

[32] Bos P. , Monden R. , Benraad C. , Groot J. , Voshaar R. O. , and Hanssen D. , Latent Profile Analysis of Biopsychosocial Measures in Older Patients with (Un)Explained Persistent Somatic Symptoms, Comprehensive Psychiatry. (2024) 135, 10.1016/j.comppsych.2023.152527. 39208557

[33] Von Korff M. , Ormel J. , Keefe F. J. , and Dworkin S. F. , Grading the Severity of Chronic Pain, Pain. (1992) 50, no. 2, 133–149, 10.1016/0304-3959(92)90154-4. 1408309

[34] Lovibond P. F. and Lovibond S. H. , The Structure of Negative Emotional States: Comparison of the Depression Anxiety Stress Scales (DASS) with the Beck Depression and Anxiety Inventories, Behaviour Research and Therapy. (1995) 33, no. 3, 335–343, 10.1016/0005-7967(94)00075-U. 7726811

[35] Preece D. , Becerra R. , Robinson K. , Dandy J. , and Allan A. , The Psychometric Assessment of Alexithymia: Development and Validation of the Perth Alexithymia Questionnaire, Personality and Individual Differences. (2018) 132, 32–44, 10.1016/j.paid.2018.05.022.

[36] Sheykhangafshe F. B. , Farahani H. , and Watson P. , Using Exploratory Graph Analysis (EGA) in Validating the Structure of the Perth Alexithymia Questionnaire in Iranians with Chronic Pain, Frontiers in Psychology. (2024) 15, 10.3389/fpsyg.2024.1400340. PMC11253556 39021647

[37] Sullivan M. J. , Bishop S. R. , and Pivik J. , The Pain Catastrophizing Scale: Development and Validation, Psychological Assessment. (1995) 7, no. 4, 524–532, 10.1037/1040-3590.7.4.524.

[38] Blevins C. A. , Weathers F. W. , Davis M. T. , Witte T. K. , and Domino J. L. , The Posttraumatic Stress Disorder Checklist for DSM‐5 (PCL‐5): Development and Initial Psychometric Evaluation, Journal of Traumatic Stress. (2015) 28, no. 6, 489–498, 10.1002/jts.22059. 26606250

[39] Cloninger C. R. , Temperament and Personality, Current Opinion in Neurobiology. (1994) 4, no. 2, 266–273, 10.1016/0959-4388(94)90092-4. 8038587

[40] Allport G. W. and Ross J. M. , Personal Religious Orientation and Prejudice, Journal of Personality and Social Psychology. (1967) 5, no. 4, 432–443, 10.1037/h0021212. 6051769

[41] Quinless F. W. and Nelson M. A. M. , Development of a Measure of Learned Helplessness, Nursing Research. (1988) 37, no. 1, 11–15, 10.1097/00006199-198801000-00003. 3340571

[42] Wu X. , Xu T. S. , Ji M. et al., Latent Profile Analysis of Pain Catastrophizing in Post-Operative Lung Cancer Patients, Journal of Pain Research. (2025) 18, 1735–1745, 10.2147/JPR.S507027. 40191622 PMC11971992

[43] Obbarius A. , Fischer F. , Liegl G. et al., A Step Towards a Better Understanding of Pain Phenotypes: Latent Class Analysis in Chronic Pain Patients Receiving Multimodal Inpatient Treatment, Journal of Pain Research. (2020) 13, 1023–1038, 10.2147/JPR.S235336. 32523372 PMC7234963

